# Ultrahigh-efficiency quantum dot light-emitting diodes

**DOI:** 10.1038/s41377-024-01481-7

**Published:** 2024-05-31

**Authors:** Lian Duan

**Affiliations:** Key Laboratory of Organic Optoelectronics and Molecular Engineering of Ministry of Education, Department of Chemistry, Tsinghua University, 100084 Beijing, China

**Keywords:** Inorganic LEDs, Quantum dots

*Nature Photonics* **18**, 186–191 (2024)

10.1038/s41566-023-01344-4

The external quantum efficiencies (EQEs) of the state-of-the-art quantum dot light-emitting diodes (QD-LEDs) are limited by the out-coupling efficiency. Orienting the transition dipole moment (TDM), or in other words, making light emission directional, is a practically efficient approach to overcome this limitation. Nanoplates, nanorods, and dot-in-plate nanocrystals offer easily controllable TDM orientation; however, their low internal quantum efficiency (IQE) offset the gain in out-coupling, leading to no improvement in EQE. A joint team of researchers from the University of Science and Technology of China, Henan University and University of Toronto has developed isotropic-shaped QDs with directional light emission that does not compromise the IQE. These QDs feature a mixed crystallographic structure, i.e. they have both wurtzite and zinc blende phases in each single nanocrystal, which allow directional light emission at a single particle level. These QD also have strong internal dipole–dipole interaction that facilitates the alignment of light emission in ensemble films. Thanks to the enhanced photon out-coupling, the LED made from these QDs represents a peak EQE of 35.6%—far above the theoretical upper limit of devices using isotropic QD emitters.

